# Mitigating the Impact of Bats in Historic Churches: The Response of Natterer’s Bats *Myotis nattereri* to Artificial Roosts and Deterrence

**DOI:** 10.1371/journal.pone.0146782

**Published:** 2016-01-15

**Authors:** Matt R. K. Zeale, Emily Bennitt, Stuart E. Newson, Charlotte Packman, William J. Browne, Stephen Harris, Gareth Jones, Emma Stone

**Affiliations:** 1 School of Biological Sciences, Life Sciences Building, University of Bristol, 24 Tyndall Avenue, Bristol, BS8 1TQ, United Kingdom; 2 British Trust for Ornithology, The Nunnery, Thetford, Norfolk, IP24 2PU, United Kingdom; 3 Graduate School of Education, and Centre for Multilevel Modelling, University of Bristol, 2 Priory Road, Bristol, BS8 1TX, United Kingdom; Università degli Studi di Napoli Federico II, ITALY

## Abstract

Bats frequently roost in historic churches, and these colonies are of considerable conservation value. Inside churches, bat droppings and urine can cause damage to the historic fabric of the building and to items of cultural significance. In extreme cases, large quantities of droppings can restrict the use of a church for worship and/or other community functions. In the United Kingdom, bats and their roosts are protected by law, and striking a balance between conserving the natural and cultural heritage can be a significant challenge. We investigated mitigation strategies that could be employed in churches and other historic buildings to alleviate problems caused by bats without adversely affecting their welfare or conservation status. We used a combination of artificial roost provision and deterrence at churches in Norfolk, England, where significant maternity colonies of Natterer’s bats *Myotis nattereri* damage church features. Radio-tracking data and population modelling showed that excluding *M*. *nattereri* from churches is likely to have a negative impact on their welfare and conservation status, but that judicious use of deterrents, especially high intensity ultrasound, can mitigate problems caused by bats. We show that deterrence can be used to move bats humanely from specific roosting sites within a church and limit the spread of droppings and urine so that problems to congregations and damage to cultural heritage can be much reduced. In addition, construction of bespoke roost spaces within churches can allow bats to continue to roost within the fabric of the building without flying in the church interior. We highlight that deterrence has the potential to cause serious harm to *M*. *nattereri* populations if not used judiciously, and so the effects of deterrents will need careful monitoring, and their use needs strict regulation.

## Introduction

Conservation is increasingly in conflict with other human activities [1,2]. Conflict can be particularly acute when species of conservation concern adopt human dwellings as nest sites or roosts, and the objectives of conservation are imposed at the expense of concerns such as the protection of cultural heritage [3]. If managed poorly, resulting negative impacts on human livelihoods and wellbeing can engender hostility towards species and undermine effective conservation [1,2].

In England, historic churches are treasured and enduring features in the landscape. Approximately 60% of pre-16th century churches are estimated to contain bat roosts and some have provided valuable roosting sites for many generations of bats. At least ten species of bat in England roost in churches [4]. While the presence of bats often goes unnoticed by people and does not result in conflict, when bats roost and fly within a church the deposition of droppings and urine can result in damage to the historic fabric of the building [5]. This is of particular concern if irreplaceable artefacts of cultural significance, such as historic monuments, wall paintings, and memorial brasses, are affected. In addition, bats can increase the cleaning burden on parishioners responsible for the care of the building. In extreme cases, large quantities of droppings can restrict the use of a church for worship and/or other community activities.

Natterer’s bats *Myotis nattereri* often roost in historic churches, and maternity colonies that can comprise >100 bats can cause acute problems between spring and autumn. The British population of *M*. *nattereri* is internationally important [6]. In 1993, six of the world’s 12 most significant hibernation sites for the species were located in England [6]. Bats previously believed to be *M*. *nattereri* in southern Europe have recently been identified as a cryptic species, making the protection of British populations even more important [7–11].

In the United Kingdom, bats are strictly protected under European and national legislation due to concerns over their conservation status. The Conservation of Habitats and Species Regulations 2010 protects all bat roosts from destruction, damage or disturbance, whether occupied or not. This legislation also places a duty on all competent authorities, including Diocesan Advisory Committees and Consistory Courts, to take adequate account of bats when works such as building restoration have the potential to damage roosts or disturb bats. In these situations striking a balance between conserving both the natural and cultural heritage presents a significant challenge. Church communities require support to reduce the impact of bats so that the needs of people can be addressed without compromising the welfare or Favourable Conservation Status of bats. The concept of ‘Favourable Conservation Status’ (FCS) is central to the EC Habitats Directive, whereby the conservation status of a species can be defined as the sum of the influences acting on the species that may affect the long-term distribution and abundance of its populations.

We investigated mitigation strategies that could be employed in churches to alleviate problems caused by bats without adversely affecting their welfare. Strategies focussed on using a combination of artificial roost provision and deterrence at churches in Norfolk, England. While we focus on maternity colonies of *M*. *nattereri*, which are of considerable conservation importance but cause damage to church features, we also include some data on *Pipistrellus pipistrellus* and *Pipistrellus pygmaeus*. We experimented with two forms of deterrence known to affect bat behaviour: ultrasonic acoustic deterrence [12] and artificial light [13,14]. We used radio-tracking to investigate the importance of churches as roosting sites for *M*. *nattereri* and to examine the response of bats to deterrents and the provision of artificial roosts. Initially, short-term applications of deterrents were used to determine the merits of each form of deterrence. Longer-term applications of deterrents were used subsequently to examine (i) if bats habituate to deterrents, and (ii) if bat welfare is compromised by prolonged use of deterrents. Using models that consider local population density and a range of negative impacts on reproductive success that might arise from exclusions, we make predictions about the impact that deterrence may have on local populations.

## Materials and Methods

### Study sites

Records of churches occupied by colonies of *M*. *nattereri* in Norfolk were obtained from Philip Parker Associates Ltd (Norfolk, England). Focus Group Meetings with stakeholder groups were organised by the Bat Conservation Trust (BCT) to establish communications with churches and to provide a platform to voice concerns about problems caused by bats, and to discuss the feasibility of potential mitigation strategies. Roost and emergence surveys were conducted at 27 churches to confirm the presence of *M*. *nattereri* colonies. Ten churches (Table 1) contained colonies of 30 or more bats and were selected for the study following consultation with church wardens and after approval by the Diocese of Norwich. All churches had medieval origins but varied in size and structure.

**Table 1:** Church sites used in this study. The figures show the number of adult female *Myotis nattereri* radio-tagged between 2011 and 2014, and the estimated size of the colony at each site.

Site	Church	Location	*n* bats radio-tagged	Estimated colony size (*n* bats)
Cley	St. Margaret	52°56ʹN, 1°2ʹE	27	70–90
Deopham	St. Andrew	52°33ʹN, 1°1ʹE	10	60–80
Great Hockham	Holy Trinity	52°29ʹN, 0°52ʹE	26	60–80
Guestwick	St. Peter	52°48ʹN, 1°3ʹE	29	60–80
Holme Hale	St. Andrew	52°37ʹN, 0°47ʹE	41	>130
Ingham	Holy Trinity	52°46ʹN, 1°32ʹE	6	>100
Salle	St. Peter & St. Paul	52°46ʹN, 1°7ʹE	40	30–40
Swanton Morley	All Saints	52°42ʹN, 0°59ʹE	37	80–100
Toftrees	All Saints	52°48ʹN, 0°48ʹE	25	>150
Wood Dalling	St. Andrew	52°47ʹN, 1°5ʹE	6	30–40

### Artificial roosts and deterrents

*M*. *nattereri* maternity colonies adopt bat boxes for roosting [15–19], and so two boxes were installed at six of the ten churches, one inside and one outside at roof height, to encourage bats to move from existing roost locations inside churches where they were causing a problem. We chose a box design that has been successful at attracting *Myotis* spp. previously [20] and installed heat mats (Habistat vivarium heat mat; Euro Rep, Middlesex, UK) and thermostats (Habistat dimming thermostat; Euro Rep, Middlesex, UK) to prevent the temperature inside the boxes falling below that favoured by *M*. *nattereri* (*circa* 22^°^C) [21]. In addition, at Holme Hale we experimented with ‘boxing in’ two major entry points used by bats accessing the church interior, so that bats entering the church via these points emerged into an enclosed roosting area that was sealed from the internal spaces of the church where droppings and urine were causing significant problems. These bespoke (i.e. custom-made) roost spaces incorporated roof timbers and mortise joints that had been used previously by the bats, and had sufficient volume to allow the bats to fly within them (S1 Appendix).

For acoustic deterrence, we used ultrasound speaker units (Deaton Engineering Inc., Georgetown, Texas, USA) previously shown to reduce bat fatalities at wind turbines [12]. Hereafter referred to as the Deaton device, the deterrent emitted continuous broadband ultrasound from 20 to 100 kHz with output of highest amplitude at 50 kHz, coinciding with the 40–50 kHz mean frequency of maximum energy of echolocation calls emitted by *M*. *nattereri* [22,23]. To minimise cost of future mitigation for churches, we developed a second acoustic deterrent in collaboration with Concept Research Ltd (Stevenage, Hertfordshire, UK): it offered substantial savings in size, weight and cost compared with the Deaton device. Hereafter referred to as the CR device, the deterrent emitted constant frequency signals between 40 and 60 kHz, oscillating between upper and lower frequencies in 3 kHz stepwise increments occurring every 4–5 seconds. We measured the sound pressure levels (SPLs) of sounds emitted by the two devices in an anechoic room at the University of Bristol using a Sanken CO-100K Super Wide Range Microphone (Sanken Microphone Co. Ltd., Tokyo, Japan). On axis, the estimated mean intensity of four Deaton speaker units was 120 dB RMS SPL at one metre. The equivalent mean intensity of three CR speakers was 90 dB RMS SPL, and so the Deaton device emitted ultrasound at amplitudes approximately 30 times higher than the CR device.

For light deterrence, we used a 400 W halogen lamp (Defender Twin 400 W 110 V Telescopic Tripod Work Light; Defender UK, Nottingham, UK). Two lamps were used in larger churches to illuminate experimental areas effectively.

### Bat capture and radio-tagging

We used radio-tracking to determine the roosting behaviour, home range areas, habitat preferences, and nocturnal activity of bats, and to examine the response of bats to deterrents. Bats were caught inside churches soon after emergence at dusk using harp traps, and as they emerged from church buildings using harp traps and hand nets. The reproductive state of bats was determined at the start of each experiment to ensure that the roost contained neither heavily pregnant nor lactating bats with dependent young [24]. Adult female bats (*n* = 247) were fitted with lightweight radio-transmitter tags (PIP Ag317, 0.47g, Biotrack Ltd, Wareham, Dorset, UK) weighing <7% of the weight of the bat using an ostomy adhesive solution (Salts Healthcare, Birmingham, UK). Tagged bats were fitted with aluminium bands (3.5 mm, Porzana Ltd, Icklesham, East Sussex, UK) to allow identification of recaptured individuals.

Experiments were performed under licence from Natural England (licence numbers: 20122211; 2014/SCI/0362). The study was approved by the University of Bristol’s Home Office Liaison Team and Ethical Review Group, and was agreed by a Project Advisory Group that included representatives from the BCT, Church Buildings Council, Defra, Ecclesiastical Architects and Surveyors Association, English Heritage, the National Trust and Natural England.

### Short-term acoustic deterrence of roosting bats

We tested the hypothesis that roosting bats could be moved by exposure to intense ultrasound. Experiments designed to examine the response of bats to four-day applications of the Deaton device were undertaken initially at six churches (Cley, Guestwick, Holme Hale, Salle, Swanton Morley and Toftrees) between 4 August and 9 September 2012, after bats had given birth and juveniles were independent. The experimental procedure is described in Table 2. Four speaker blocks were positioned inside churches directly below 1–2 roosts that were occupied by most or all of the radio-tagged bats prior to the start of the deterrent period. The vertical distance from speaker to roost was standardised across sites (mean 9.5 ± 0.7 metres). The speakers emitted a loud continuous sound that included some frequencies within the audible spectrum for humans and so were switched off during the day to avoid disturbing visitors to churches. We radio-tagged 17 adult female bats at Cley, 14 at Guestwick, 16 at Holme Hale, 11 at Salle, 14 at Swanton Morley and 15 at Toftrees. All bats were located during the day using a R1000 receiver (Communications Specialists Inc., Orange, CA, USA) and a 3-element Yagi antenna to identify day roosts and to monitor roost movements. At night, locational fixes were recorded every 5–10 minutes between dusk and dawn using the ‘homing-in’ method [25–28] to examine foraging behaviour. Any night with less than 90% contact time with a bat was excluded from the final analyses as the complete pattern of movements throughout the night could not be identified. Droppings that accumulated below roosts where deterrents were installed were collected each day, oven dried to constant weight, and the dry mass of daily samples recorded.

**Table 2:** Experimental procedure to examine the response of *Myotis nattereri* to short-term applications of ultrasonic acoustic and artificial lighting deterrence inside churches.

Day	Period	Activity
1	Trapping	Adult female bats caught and radio-tagged.
2–5	Control	Deterrent installed on day 2 but remains switched off to control for effect of physical presence of deterrent on bat behaviour. Data from first night of control period removed from analyses due to effect of disturbance caused by trapping and tagging on the previous evening.
6–9	Deterrent	Day 6 deterrent switched on at midnight after bats emerged from the church. On days 7–9 deterrent switched on before emergence and switched off at dawn after bats returned to day roosts.
10–13	Post-deterrent	Deterrent switched off at dawn on day 10 and removed from the church.

Home range areas for radio-tracked bats were calculated after plotting radio fixes in ArcGIS 10 (Esri Inc., Redland, CA, USA). Fix data were imported into Ranges 7 (Anatrack Ltd, Wareham, Dorset, UK) and used to calculate colony home ranges (100% minimum convex polygons; MCPs) and core foraging areas (cluster cores) [27,28]. Analysis of utilisation distribution discontinuities showed that up to 20% of fix locations increased the size of foraging areas disproportionately and an examination of these fixes revealed that they were primarily recorded as bats commuted between roosts and foraging areas. Thus, 80% cluster cores were used to define core foraging areas.

Habitat preferences were examined by comparing the habitat composition of areas in which each bat foraged (80% cluster cores) to that available (individual MCP home ranges) [27–29]. Compositional analysis (Compositional Analysis Plus Microsoft Excel tool 6.2, Smith Ecology Ltd, Abergavenny, Gwent, UK) was used to determine whether habitats were used in line with availability or if selection was occurring, and to determine the ranking of habitat types [30]. Habitat data were extracted from digital maps developed in-house using ArcGIS 10 using the five broad habitat categories described in S1 Table.

To examine whether the response of bats to acoustic deterrence differed in spring, when bats were in early stages of pregnancy, from that recorded in summer i.e. after bats had given birth, experiments using the Deaton device were repeated at four churches (Guestwick, Holme Hale, Swanton Morley and Great Hockham) between 15 and 26 May 2014. We radio-tagged 15 adult female bats at Great Hockham, 8 at Guestwick, 15 at Holme Hale and 13 at Swanton Morley, and located them each day to monitor roost movements.

To examine whether bats could be moved from roosts using the low-cost CR device, which emitted ultrasound at lower amplitudes than the Deaton device, we tested the CR device at two churches, Salle and Toftrees, between 7 August and 13 September 2013 following the same experimental procedure used for the Deaton device (Table 2). In each experiment, to compensate for the lower intensity signal produced by the CR device, we installed three speaker units approximately one metre from roost entrances. All units pointed directly at roost entrances and, where possible, up into roost cavities. The number of bats emerging from the roost inside the church where the CR devices were located was recorded each evening by an observer on the ground using a Batbox III D heterodyne bat detector (Batbox Ltd., Steyning, West Sussex, UK) and a night vision monocular (Yukon Advanced Optics Worldwide, Vilnius, Lithuania).

### Long-term acoustic deterrence of roosting bats

We tested the hypothesis that bats did not habituate to the ultrasonic deterrent at three churches (Deopham, Guestwick and Swanton Morley) between 26 July and 12 September 2013. The Deaton device was used in the same way as that described for short-term experiments but for 15 days. We radio-tagged 10 adult female bats at Deopham, 7 at Guestwick and 10 at Swanton Morley, and located them each day to monitor roost movements. Emergence surveys were undertaken every second evening at dusk. The number of bats that exited from the church and the number of bats that emerged from the roost inside the church where the Deaton device was located were recorded by observers on the ground using heterodyne bat detectors and night vision monoculars.

### Deterrence of roosting bats using artificial lighting

We tested the hypothesis that bats could be moved from roosts using artificial lighting at one site, Salle, between 26 July and 2 August 2012 following the experimental procedure described in Table 2. One 400 W halogen lamp was positioned 7.5 metres below the roost used by the majority of radio-tagged bats and the lamp was directed upward to illuminate roost exits. Eleven bats were fitted with radio-tags and the response of the bats to light was recorded in the same way as for short-term acoustic deterrence experiments using the Deaton device.

### Creation of bat ‘no-fly zones’ using artificial lighting

We tested the hypothesis that bats could be excluded from large areas within churches at four sites (Cley, Great Hockham, Holme Hale and Salle) between 25 July and 12 September 2013 following the experimental procedure described in Table 2. One or two 400 W halogen lamps were used in a directed way to raise ambient light levels in the chancel (the part of a church near the altar reserved for the clergy and choir) while keeping all other areas of the church in relative darkness. Light levels at roosts and at exit points from the church that were used by bats were kept to a minimum. Bat activity was monitored in the chancel (‘lit zone’) and in the opposite end of the church (‘dark zone’) using Anabat automated frequency division bat detectors (Titley Scientific, Columbia, MO, USA) that recorded bat activity throughout the night, and using infrared digital cameras (Y-cam Cube HD 720; Y-cam Solutions Ltd, Richmond, Surrey, UK) that recorded bat activity for two hours after sunset. We classified bat detector records according to three species/groups: common pipistrelle *Pipistrellus pipistrellus*, soprano pipistrelle *Pipistrellus pygmaeus* and *Myotis* spp. We used the group *Myotis* spp. because echolocation calls of *M*. *nattereri* are not readily distinguishable from those of other species of the genus *Myotis*, although most records for this group were expected to be *M*. *nattereri*. We radio-tagged 10 adult female *M*. *nattereri* at Cley, 11 at Great Hockham, 10 at Holme Hale and 10 at Salle. The emergence time of radio-tagged bats from churches was recorded each night and the locations of roosting bats were recorded each day to monitor roost movements. Light levels (illuminance in lux) were measured at the centre of lit and dark zones using a Konica Minolta T-10 illuminance meter (Konica Minolta Inc., Tokyo, Japan); this is sensitive to illuminance as low as 0.01 lux and was held vertically at a height of 1.7 m above ground level and oriented towards the lamps.

### Statistical analysis

To test the hypothesis that the roosting behaviour of bats was affected by short-term applications of the Deaton device, we employed an event history-type modelling process to investigate the probability of an event occurring i.e. the movement of a bat each day throughout the experiment. We identified three types of roost, namely ‘original’ (the roost affected by the deterrent during the deterrent period), ‘alternative’ (a roost inside the church but away from the deterrent), and ‘outside’ (any roost not inside the church), and categorised the responses of each bat at each church. The movement of bats over each day of the experiment was identified by linking the roost location of a bat on one day to its location on the previous day.

We fitted two multistate models to the transition data to determine (i) whether bats were deterred from the original roost inside the church, and (ii) whether bats were deterred from roosting anywhere inside the church i.e. were forced outside. For the first model, the roost categories ‘alternative’ and ‘outside’ were merged and transition states were reduced to two such that the roost categories became ‘original’ and ‘other’ and the response of bats became either to ‘move’ from or to ‘stay’ at a roost. For the second model, transition states were reduced as in the first model but ‘original’ and ‘alternative’ roost categories were merged to give the categories ‘inside’ and ‘outside’ the church. The aim was to determine whether the response of bats to ‘move’ or to ‘stay’ differed significantly according to two explanatory fixed effect variables: roost type (i.e. the category of roost that the bat was in) and period (i.e. control, deterrent or post-deterrent). All statistical modelling was performed in MLwiN v2.1 [31].

We tested hypotheses regarding whether spatial behaviour, emergence and foraging time were affected by acoustic deterrence. To determine if individual home ranges (100% MCPs), core foraging areas (80% clusters), maximum range spans (distance from roost to furthest edge of cluster core foraging area), time of emergence, time of return, and time spent foraging were affected by short-term applications of the Deaton device, we fitted a series of general linear mixed models (GLMMs) to each of these response variables. Data for individual home ranges, core foraging areas and time of emergence were log-transformed prior to fitting the models. The explanatory variables tested in each model included two categorical fixed effects (site (*n* = 6) and period (control versus deterrent)) along with the interactions between site and period to investigate site-dependent effects of the deterrent. Nightly measurements were nested within bats, and so we fitted bat identity as a random effect to control for dependence within bats.

To test the hypothesis that the Deaton device affected whether individual bats foraged in different locations during control versus deterrent periods, we calculated core foraging areas for each bat-night and recorded the percentage overlap of foraging areas for 356 pairs (76 control-control pairs, 69 deterrent-deterrent pairs, 211 control-deterrent pairs) from 34 bats across six sites. To determine whether foraging site overlap (response variable) was affected by the presence of the deterrent, we fitted a multiple membership multiple classification (MMMC) model with MCMC estimation [32] to the data because each overlap measurement was nested within a pair of nights within a bat. Explanatory fixed effect variables included site, comparison type (control-control, deterrent-deterrent, control-deterrent) and time interval (time (*n* days) between nights within each night-pair). No effect of site was found and so site was removed from the model.

To test the hypothesis that responses of bats to the Deaton device differed in spring and summer i.e. pre- and post-natal periods, we fitted multistate models to the combined spring and summer data and included parameters in the model to capture differences in probabilities of each type of roost movement being made between the two periods using Wald (chi-square) tests. Variability is described throughout as standard deviations (SD) of the mean.

### Population models

Currently it is not possible to assess how exclusions resulting from deterrence might affect the Favourable Conservation Status of *M*. *nattereri* because we do not know the critical population parameters to measure. So we developed a stochastic matrix population model that described *M*. *nattereri* demography and provided a method whereby changes in productivity i.e. number of female young reared and age-specific survival, could be simulated to examine the effects on population growth. Since a thorough review of these techniques has been published elsewhere [33], we present a summary of the principles involved in formulating the model. Additional explanations are provided in S2 Appendix.

We constructed a stochastic matrix population model for *M*. *nattereri* at the end of the breeding season. We assumed that the sex ratio was equal and modelled only for females as these comprise the majority of bats in most maternity colonies. There were three age classes in the model: female young produced by the end of a breeding season, females in their second calendar year (their first breeding season), and females in their third calendar year or older (their second or subsequent breeding season). We introduced stochastic variation in age-specific survival to assess the effects of random year-to-year variation in life-cycle parameters. Since there was a lack of information on annual variation in litter size and proportion of females breeding, we assumed that these variables were constant rather than stochastic. While density-dependent factors may be important, they were not considered because any influence on population growth rate was unknown. In the absence of information on movements from outside the local population, we assumed that populations were closed i.e. there was no immigration or emigration.

The vital rates used for *M*. *nattereri* are summarised in Table 3. Additional information on how the vital rates were derived is provided in S2 Appendix. The starting population (colony) was 100 females, chosen to represent a typical colony size, and distributed according to the stable age distribution of the equivalent deterministic model. 1000 realisations were run for an arbitrary time frame of 500 years. We recorded the mean stochastic growth rate and the proportion of extinct trajectories at the end of the simulation, with an extinction threshold of 1. Matrix calculations were conducted using the program ULM [34].

**Table 3:** Vital rates used in population matrix models for *Myotis nattereri*.

	Vital rate	Estimate (SE)	Source
*Annual survival*			
Survival in first year	*S*_*1*_	0.491 (0.088)	[35]
Survival in second year	*S*_*2*_	0.684 (0.151)	[35]
Survival in third year plus	*S*_*3*_	0.875 (0.118)	[35]
*Productivity*			
Mean litter size in second year	*L*_*2*_	1.000	[36]
Mean litter size in third year plus	*L*_*3*_	1.000	[36]
Proportion breeding in second year	*Alpha*_*2*_	0.280	this study
Proportion breeding in third year plus	*Alpha*_*3*_	0.730	this study

## Results

### Use of churches by *Myotis nattereri*

Bats roosted predominantly within the church buildings they inhabited, most often among exposed roof timbers, and only occasionally used alternative roosts outside the church. Prior to the deployment of deterrents, we collected 775 records of day roosts from 247 bats at 10 churches; 84 (10.8%) records were of bats roosting outside the church, typically in trees close to foraging grounds. Inhabited dwellings and uninhabited buildings such as sheds and garages were rarely used. We only found one alternative roost that we considered was capable of supporting all bats from the maternity colony, at Cley, where the colony in the church comprised an estimated 70 to 90 bats. The roost was in a large uninhabited outbuilding 1.3 km from the church and adjacent to a large area of preferred foraging habitat used by radio-tagged bats, and was used repeatedly by five radio-tagged bats. Within churches, bats roosted in multiple locations at ceiling height and frequently moved between roosts. Whole colonies typically changed roost once per week.

**Fig 1:**
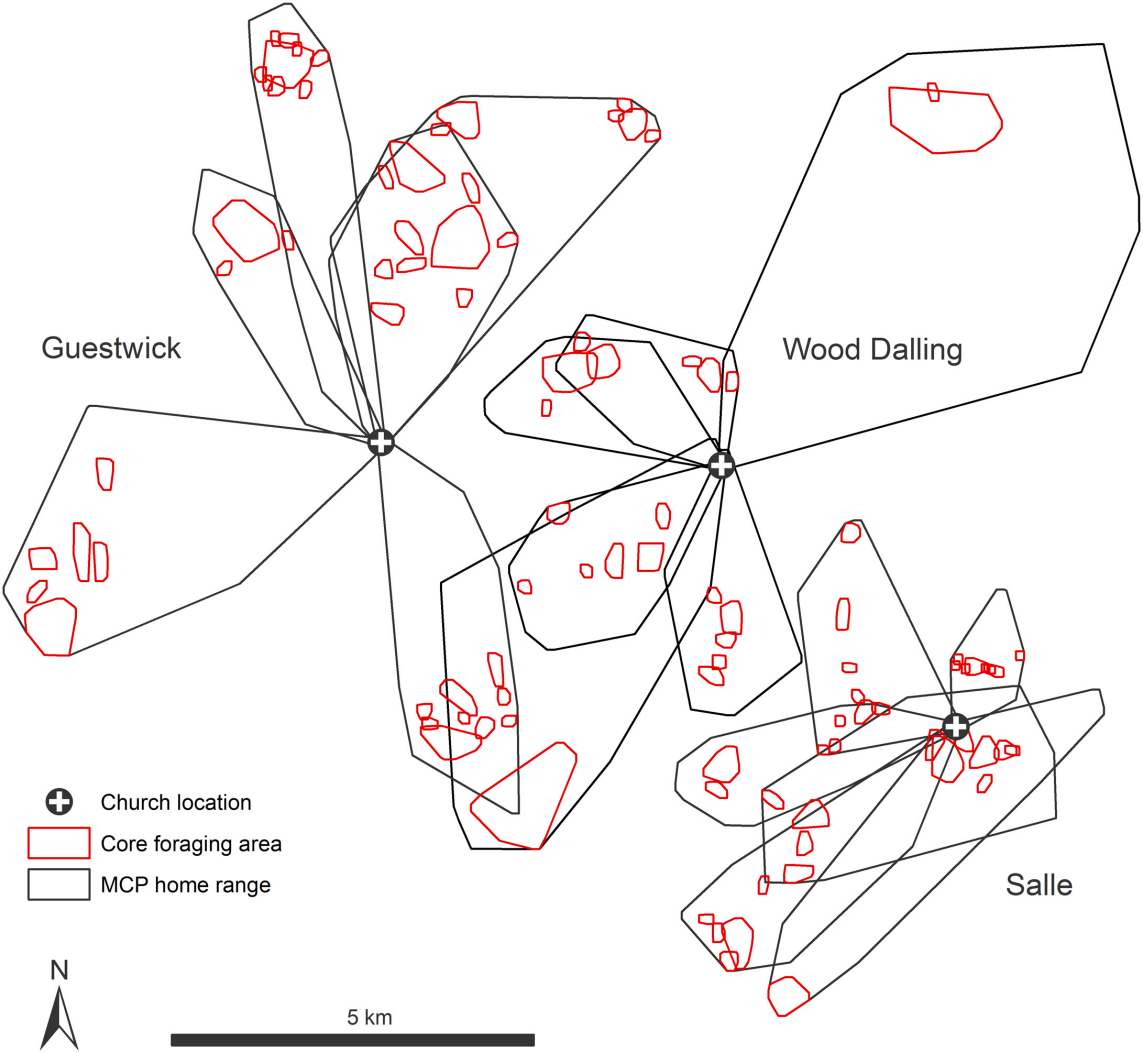
Examples of individual bat home range areas and core foraging areas from 18 adult female *Myotis nattereri*. The black polygons denote 100% MCP home ranges and the red polygons 80% cluster cores. Data are from 18 bats radio-tracked at three churches (Guestwick, Salle and Wood Dalling; *n* = 6 bats per site).

We collected data on foraging from 48 bats followed as focal individuals at eight sites (*n* = 6 bats per site). Home range areas of bats ranged from 130.9 ha to 2468.7 ha (Table 4). On average bats travelled 4.0 ± 1.4 km (range 1.4–7.7 km) from roosts to foraging areas and used a small fraction (9.6 ± 4.1%) of their home range area specifically for foraging. These ‘core foraging areas’ were on average 63.6 ± 41.8 ha in size. Individual bats were faithful to exclusive foraging patches i.e. there was little or no overlap of core foraging areas between bats. Adjacent colonies also showed exclusive and non-overlapping ranges (Fig 1).

**Table 4:** Home range areas (100% minimum convex polygons), core foraging areas (80% clusters cores) and range spans (mean maximum nightly distance from roost to centroid of cluster core foraging area) for 48 adult female *Myotis nattereri* (*n* = 6 bats per site). Figures are means ± SD (range).

Site	Home range area (ha)	Core foraging area (ha)	Max. range span (km)
Cley	369.2 ± 63.3(279.5–456.7)	44.9 ± 10.4(34.0–62.4)	2.7 ± 0.7(1.6–3.3)
Guestwick	757.6 ± 280.4(419.7–1138.1)	74.4 ± 33.0(45.9–134.1)	5.0 ± 0.9(3.9–5.9)
Holme Hale	483.3 ± 286.1(194.5–878.6)	33.7 ± 23.0(12.3–54.0)	3.2 ± 0.8(2.4–4.2)
Ingham	1186.6 ± 703.9(557.5–2468.7)	107.4 ± 50.8(56.1–193.4)	4.7 ± 1.9(2.5–7.7)
Salle	484.59 ± 18.51(130.9–754.5)	36.1 ± 18.5(13.6–65.3)	3.5 ± 1.2(1.4–4.7)
Swanton Morley	702.0 ± 318.3(357.6–1228.9)	64.9 ± 35.3(24.5–106.9)	3.8 ± 0.8(3.1–5.0)
Toftrees	869.7 ± 651.7(324.1–1968.9)	74.8 ± 60.3(32.3–194.0)	4.6 ± 1.4(2.9–6.6)
Wood Dalling	880.0 ± 810.5(345.6–2345.5)	63.0 ± 41.5(29.2–119.8)	4.0 ± 1.7(2.7–6.4)

Bats preferred to forage in woodland, which on average comprised only 11% of available land cover within home ranges, followed by grassland (Table 5). Arable and riparian habitats, including fresh water, marsh, wet woodland and grassland, were not preferred. Overall, built-up habitat, defined here as areas of low to medium density rural residential land cover (<40% cover) was least preferred; there were no heavily urbanised or industrial areas.

**Table 5:** Habitat preferences exhibited by *Myotis nattereri* at eight maternity colony sites (*n* = 6 bats per site). Habitat categories to the left of > were selected over those to the right, with >>> showing a significant difference between adjacent habitat types; *P*-values <0.05 show that the selection of habitat types was non-random.

Site	Ranked habitat types									*P*
Cley	Woodland	>>>	Pasture	>	Arable	>	Riparian	>	Built-up	<0.05
Guestwick	Woodland	>	Pasture	>	Arable	>	Built-up	>	Riparian	<0.05
Holme Hale	Woodland	>	Pasture	>	Riparian	>	Arable	>	Built-up	<0.01
Ingham	Woodland	>	Pasture	>	Arable	>	Riparian	>	Built-up	<0.01
Salle	Woodland	>	Pasture	>	Built-up	>	Riparian	>	Arable	<0.001
Swanton Morley	Woodland	>	Pasture	>	Riparian	>	Arable	>	Built-up	<0.001
Toftrees	Woodland	>	Pasture	>	Riparian	>	Arable	>	Built-up	<0.05
Wood Dalling	Woodland	>	Pasture	>	Arable	>	Riparian	>	Built-up	<0.01

Bats emerged 85 ± 38 minutes after sunset, foraged for a total of 373 ± 57 minutes, and returned 114 ± 37 minutes before sunrise (*n* = 48 bats). While bats occasionally night-roosted either inside or outside churches (recorded in 12 of 121 bat-nights), foraging was typically focussed into a single foraging bout. Night-roosting events lasted on average 27 ± 13 minutes.

### Short-term acoustic deterrence of roosting bats

**Fig 2:**
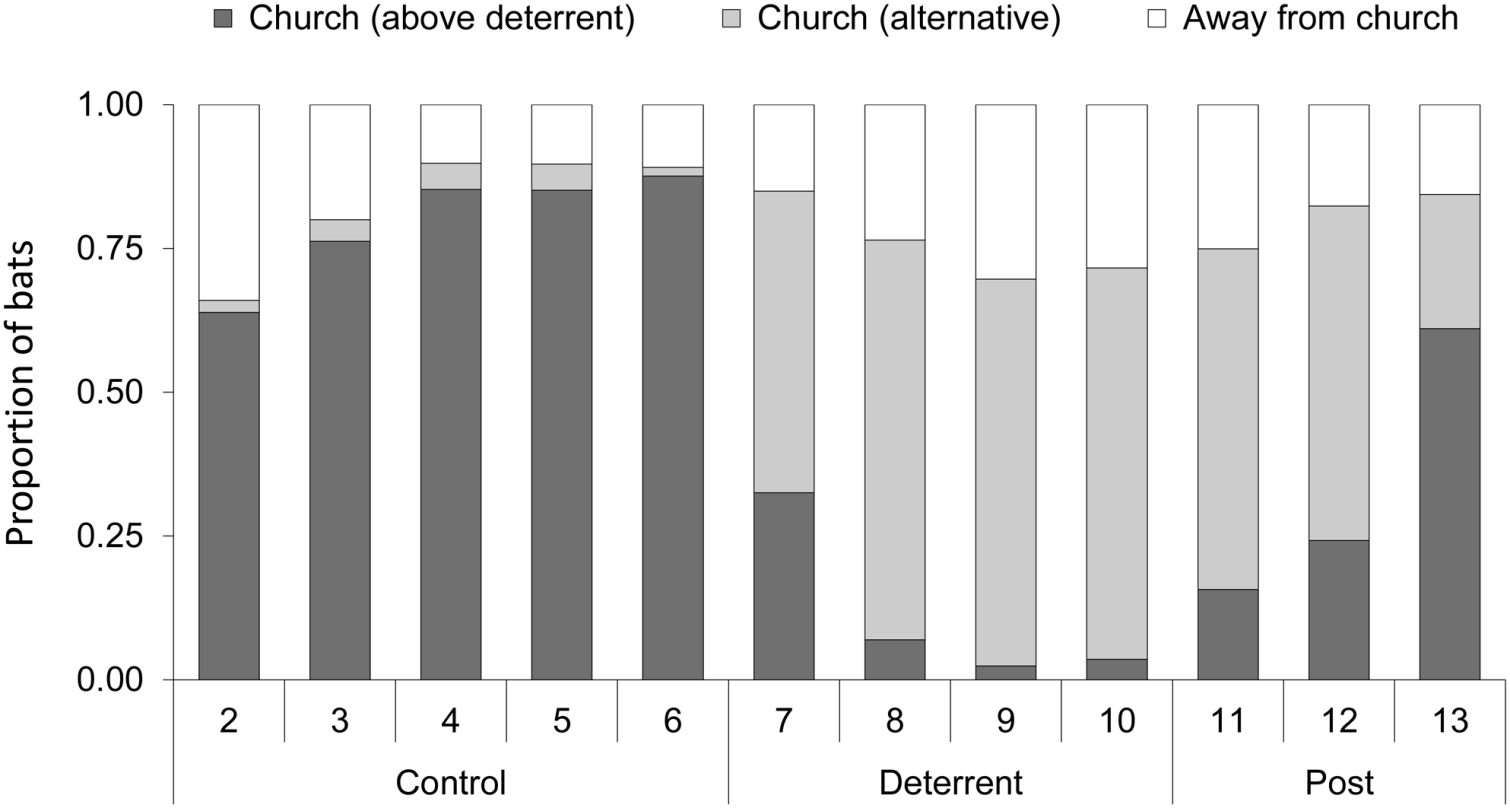
Response of *Myotis nattereri* to short, four-day applications of the Deaton (ultrasound) device. The figure shows the mean (*n* = 6 sites) proportion of radio-tagged bats (*n* = 87) roosting each day in the original roost above the deterrent, at an alternative roost within the church, or at an alternative roost outside the church during control (deterrent off), deterrent (deterrent on) and post-deterrent (deterrent off) periods. The bats were radio-tagged on day 1.

*M*. *nattereri* was deterred from using roosts by the Deaton device (Fig 2). On average (*n* = 6 sites), bats were faithful to the original roost during the control period (mean 94% probability of returning to the roost each day) (Table 6). During the deterrent period, initially some bats continued to use the original roost (37% probability of returning) but, once a bat left, it was highly unlikely that it would return either from an alternative roost inside the church (1% probability) or from outside (3% probability), and on average it took 1–2 days for all bats to be deterred. After the deterrent was removed, the probability that bats returned to the original roost increased and bats that returned typically then stayed in the original roost (87% probability). However, most bats did not return to the original roost but continued to roost in alternative roosts inside (77% probability of remaining in an alternative roost) or outside the church (71% of remaining outside). During the deterrent period, radio-tagged bats at Holme Hale and Toftrees roosted outside considerably more often, whereas at all other churches the bats continued to roost predominantly inside the church but in alternative roosts away from the deterrent. When we fitted multistate models to transition data, in both models we found that both roost type and period (i.e. control, deterrent, post-deterrent) contributed significantly to explaining variation in the probability of changing roost (S2 Table), indicating that the presence or absence of a deterrent significantly affected the movements of bats to and from specific roost locations.

**Table 6:** Probability matrices showing the mean (*n* = 6 sites) probability of *Myotis nattereri* moving between different roost ‘states’ during control (deterrent off), deterrent (deterrent on) and post-deterrent (deterrent off) periods. ‘Original’ = the roost affected by deterrent during the deterrent period, ‘alternative’ = a roost inside the church not directly affected by the deterrent, ‘outside’ = any roost not inside the church. ‘Post’ refers to post-deterrent period.

Period	Move from	Move to			*n* records
		original	alternative	outside	
	original	0.94	0.01	0.05	196
Control	alternative	0.45	0.45	0.10	9
	outside	0.50	0.00	0.50	34
	original	0.37	0.49	0.14	99
Deterrent	alternative	0.01	0.91	0.08	115
	outside	0.03	0.13	0.84	63
	original	0.87	0.13	0.00	30
Post	alternative	0.21	0.77	0.02	85
	outside	0.11	0.18	0.71	45

At three sites (Guestwick, Holme Hale and Swanton Morley), the accumulation of bat faeces below the original roost reduced substantially by 93.8 ± 6.1% (*n* = 3 sites) by the final day of deterrence. At two sites, Cley and Salle, bats occupied roosts located above ledges or in ceiling voids, and so droppings at ground level were negligible. At Toftrees, which was the smallest church in the study but was occupied by the largest colony of bats (Table 1), many of the droppings that accumulated below roosts originated from bats flying inside the church at night, and so the number of droppings recorded below the original roost did not differ substantially between control and deterrent periods despite bats moving from the roost in response to deterrence.

We found a significant effect of site for all foraging variables that we tested i.e. bat foraging behaviour was site-dependent; however, no effect of deterrent (Deaton device) was found for any of the variables tested (Table 7). When interactions between site and period were modelled, we found a significant effect for time of emergence only (Table 7). More specifically, the presence of the deterrent at one church, Salle, caused bats to emerge earlier than during the control period, although mean emergence time for bats at Salle during the deterrent period (72 ± 30 minutes, *n* = 18) was similar to the mean across all sites (92 ± 47 minutes, *n* = 84), while emergence time during the control period (141 ± 44 minutes, *n* = 18) was considerably later than the mean (83 ± 35 minutes, *n* = 86).

**Table 7:** Foraging responses of radio-tagged *Myotis nattereri* at 6 church sites (*n* = 6 bats/site) and effect of short-term applications of acoustic deterrence at roosts inside churches. Response variables were time of emergence, time of return to church after foraging, total time spent foraging, individual bat home range area (MCP), core foraging area (cluster core), and range span (distance from roost to furthest edge of cluster core foraging area).

Response	Fixed effects (chi-square (degrees of freedom))					
	Site	*P*	Deterrent	*P*	Interaction	*P*
Log emergence	14.24 (5)	*	0.12 (1)	ns	32.17 (5)	***
Return time	32.48 (5)	***	1.60 (1)	ns	9.65 (5)	ns
Foraging time	25.56 (5)	***	0.92 (1)	ns	8.14 (5)	ns
Log MCP	19.22 (5)	*	0.10 (1)	ns	4.01 (5)	ns
Log cluster core	15.99 (5)	*	3.06 (1)	ns	5.64 (5)	ns
Range span	26.19 (5)	***	0.02 (1)	ns	6.65 (5)	ns

* *P* < 0.05,

*** *P* < 0.001,

ns = not significant

When we examined foraging area overlap among bat-nights there was a negative correlation between the time period between pairs of nights and overlap i.e. nights that were close together had greater overlap. The random effects for bats and the pairs of nights within each bat both contributed significantly to explaining overlap data but comparison type, i.e. control-control, deterrent-deterrent or control-deterrent pairs of foraging areas did not, indicating that individual bats may shift the focus of their foraging effort to different areas within patches on a nightly basis, perhaps to maintain encounter rates with prey by avoiding foraging in areas that were exploited during previous nights. However, this effect was not influenced by the presence of a deterrent at roosts.

Habitat preferences of bats were unaffected by deterrent use. Woodland, followed by pasture, were the most preferred habitat types during both control and deterrent periods across all sites (Table 8).

**Table 8:** Habitat preferences of *Myotis nattereri* (*n* = 6 bats per site) radio-tracked at five churches during control and deterrent periods in response to ultrasound acoustic deterrence. Habitat categories to the left of > are selected over those to the right, with >>> showing a significant difference between adjacent habitat types; *P*-values <0.05 show that the selection of habitat types is non-random.

Site	Period	Ranked habitat types									*P*
Cley	Control	Woodland	>>>	Pasture	>	Arable	>	Riparian	>	Built-up	<0.05
	Deterrent	Woodland	>	Pasture	>	Riparian	>	Built-up	>	Arable	<0.01
Guestwick	Control	Woodland	>	Pasture	>	Arable	>	Built-up	>	Riparian	<0.05
	Deterrent	Woodland	>>>	Pasture	>	Arable	>	Built-up	>	Riparian	<0.001
Salle	Control	Woodland	>	Pasture	>	Built-up	>	Riparian	>	Arable	<0.001
	Deterrent	Woodland	>	Pasture	>	Arable	>	Built-up	>	Riparian	<0.001
Swanton Morley	Control	Woodland	>	Pasture	>	Riparian	>	Arable	>	Built-up	<0.001
	Deterrent	Woodland	>>>	Pasture	>	Riparian	>	Arable	>	Built-up	<0.001
Toftrees	Control	Woodland	>	Pasture	>	Riparian	>	Arable	>	Built-up	<0.05
	Deterrent	Woodland	>	Pasture	>	Riparian	>	Arable	>	Built-up	<0.01

In spring there was a significantly higher probability that bats moved from the original roost during both control (Wald χ^2^ (1) = 7.25, P < 0.001) and deterrent periods (Wald χ^2^ (1) = 5.58, P < 0.05) compared to summer, indicating that adult female bats show greater fidelity to roosts in summer when juvenile bats are present. In all other respects, the roosting behaviour of bats in spring and summer, and the response of bats to deterrents in spring and summer, were the same.

We observed a similar response by bats to the CR device as that described for the Deaton device. At Salle, the number of bats using the original roost fell by 97% after a single day of deterrence using the CR device, from 92 to three bats, and remained low throughout the deterrent period (range 3–9 bats; *n* = 4 days). At Toftrees the number of bats fell initially by 83%, from 162 bats to 28 bats, but then increased to 84 bats on the fourth day of the deterrent period. When the speaker units were removed at Toftrees, we found that droppings and urine had accumulated in the conical speaker wells, which may have affected signal intensity.

### Long-term acoustic deterrence of roosting bats

**Fig 3:**
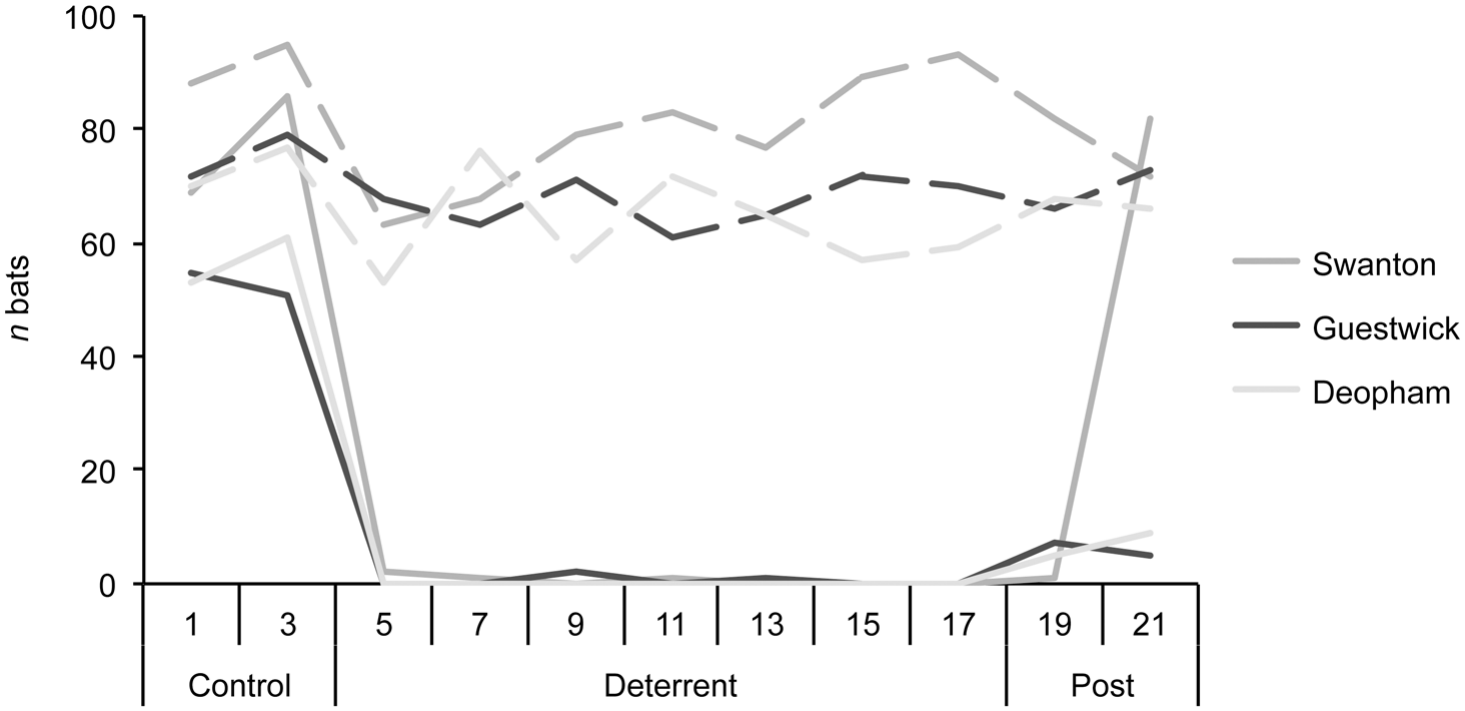
Response of *Myotis nattereri* to long-term applications of the Deaton (ultrasound) device. The figure shows the number of bats roosting inside the church (dashed lines) and in the roost above the deterrent (solid lines) each day at three churches during the control (deterrent off), deterrent (deterrent on) and post-deterrent (deterrent off) periods.

We observed no evidence of habituation to the Deaton device. Bats continued roosting in churches in similar numbers throughout the experimental period, though the bats moved away from the roost exposed to the speaker. Hence prolonged use of acoustic deterrence inside a church did not result in bats being excluded from the building, but it was effective for moving bats away from specific sites (Fig 3).

### Deterrence of roosting bats using artificial lighting

After an initial control period of five days, lights were switched on at midnight. Almost all *M*. *nattereri* flying in the church returned immediately to the illuminated roost. The following morning, all radio-tagged bats (*n* = 11) were in the roost. The following evening, when lights were switched on at dusk before bats emerged, no bats emerged from the roost. During the next deterrent night only two bats emerged and both bats returned to the roost after foraging. At this stage, lights were switched off and the experiment was terminated prematurely due to concerns over the welfare of the bats. The following night, in the absence of lights, all radio-tagged bats emerged from the roost to forage, but substantially earlier than during the control period, suggesting that they were energetically stressed. On the next night without lights, emergence times were similar to those recorded during the control period. Experiments using light in this way were not repeated.

### Creation of bat ‘no-fly zones’ using artificial lighting

**Fig 4:**
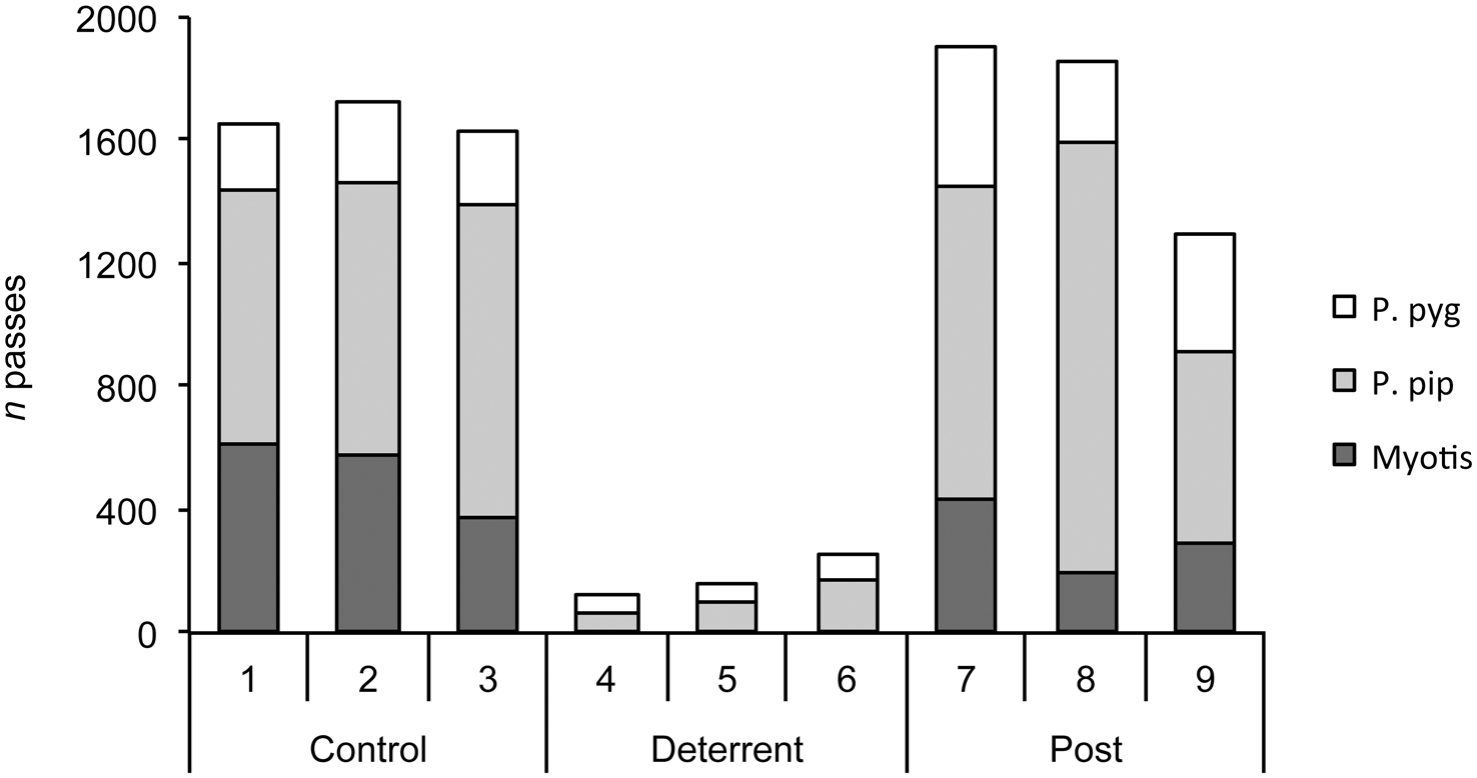
Response of bats to artificial lighting in churches. The figure shows the total number of bat passes (*n* = 4 sites) recorded each night in experimental lit zones inside churches during control (ambient light), deterrent (artificial light) and post-deterrent (ambient light) periods. P. pyg = soprano pipistrelle *Pipistrellus pygmaeus*; P. pip = common pipistrelle *P*. *pipistrellus*; Myotis = *Myotis nattereri*.

**Fig 5:**
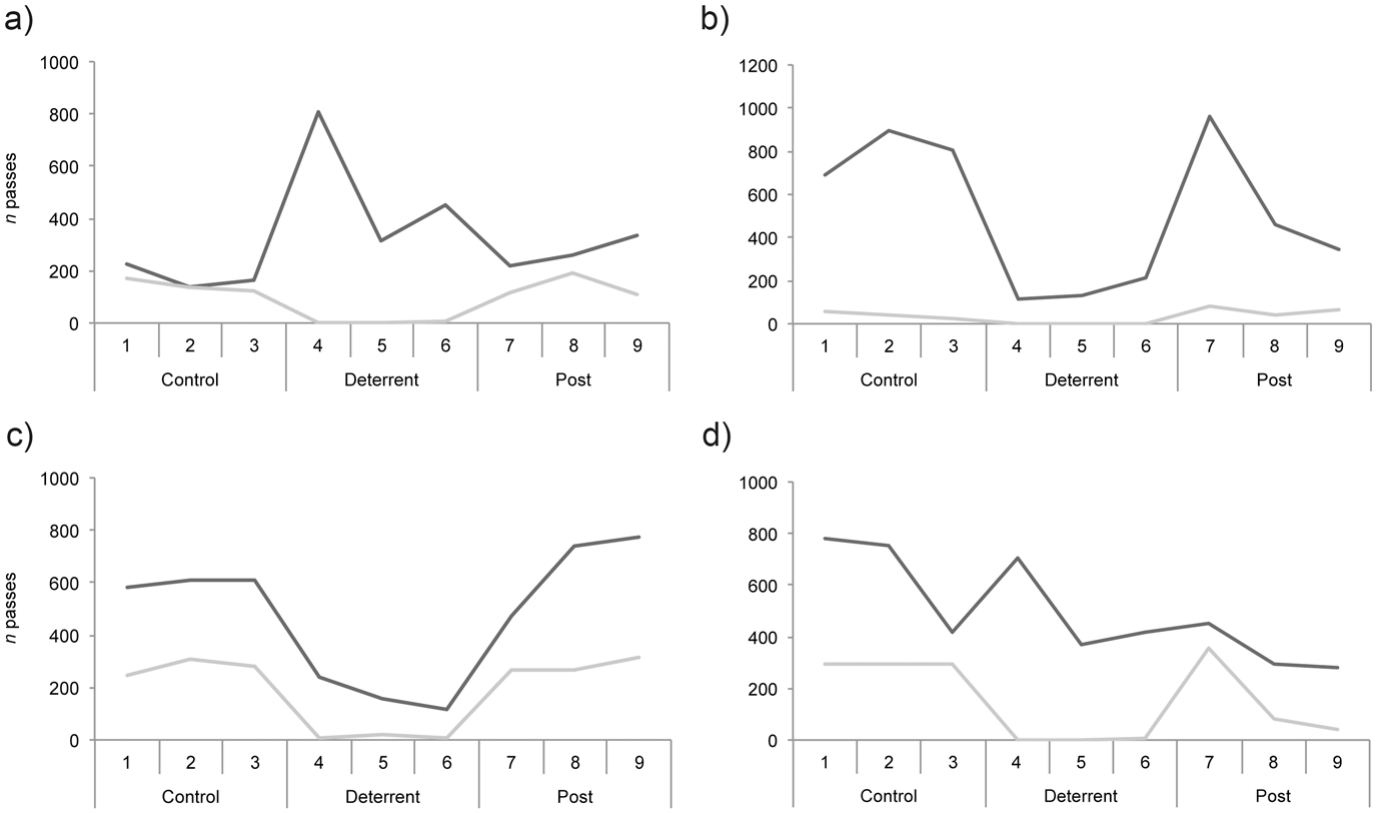
Activity of *Myotis nattereri* in lit and unlit areas of churches during lighting deterrence experiments. The figure shows the number of bat passes recorded each night for two hours after sunset using infrared video in lit (light grey lines) and unlit (dark grey lines) zones inside churches during control (ambient light), deterrent (artificial light) and post-deterrent (ambient light) periods at four churches: (a) Cley, (b) Great Hockham, (c) Holme Hale and (d) Salle.

During control periods, light levels throughout churches were <0.1 lux. When lights were switched on, the mean ambient light level recorded in the chancel (lit-zone) was 167.0 ± 96.75 lux (range 72.3–273.0). The number of bat passes in lit-zones reduced substantially on nights when the lights were switched on. Few passes by *Pipistrellus* spp. were recorded and passes by *M*. *nattereri* reduced to zero, or near to zero, at all sites (Fig 4). In dark-zones (i.e. areas away from the experimental lights) we observed a mixed response by bats during deterrent periods. Activity reduced at two churches (Great Hockham and Holme Hale), increased at one church (Cley), and was stable at another (Salle) (Fig 5). At Great Hockham and Holme Hale, the distance between dark and lit zones (range 15.5–15.9 m) was half that for Cley and Salle (range 31.3–31.7 m), and light levels in dark zones were visibly higher (1.0–1.4 lux versus 0.3–0.4 lux). So light spill at Great Hockham and Holme Hale may have been sufficient to deter some bats from using the dark zones.

At all churches the emergence time of radio-tagged bats was later and more variable during deterrent periods (mean 162 ± 203 minutes after sunset; range 49–304 minutes) than during control periods (mean 86 ± 46 minutes after sunset; range 46–117 minutes), despite our efforts to ensure that neither roosts nor bat exit points from churches were illuminated. Although all bats exited churches at some stage during deterrent periods, we recorded 23 incidences (19% of bat-nights) when a bat remained in the church all night. This represents a considerable reduction in foraging time among radio-tagged bats, and bats that remained in churches may have become energetically stressed.

### Artificial roosts

During experimental trials, bats were not recorded using the bat boxes that we provided for them. Small accumulations of droppings below some boxes inside churches were observed after experimental trials, suggesting occasional use. At Holme Hale, when major entry points used by bats to access the interior of the church were ‘boxed-in’ to develop new roost spaces that restricted access to the main interior of the church, bats used these spaces frequently. Of 88 bats that occupied the church at the start of the trial, up to 46 bats used the boxed-in areas each day, and up to 28 bats adopted an external roost location in the porch as a new roost site. As a result, the proportion of bats roosting within the church, and the associated deposition of droppings and urine in the church, reduced considerably.

### Population models

The projection matrix model derived from the vital rates in Table 3 gave a mean stochastic population growth rate λ_s_ of 0.986 i.e. a slow decline but close to stable. Survival from the third year plus (*S*_*3*_) was by far the most important parameter contributing to population growth (Table 9). Individual components of productivity i.e. mean litter size of bats breeding in their second year (*L*_*2*_), and third year plus (*L*_*3*_), and the proportion of individuals breeding in their second year (*Alpha*_*2*_) and third year plus (*Alpha*_*3*_), have comparatively small elasticities. Therefore, changes in these parameters are likely to have a comparatively small effect on population growth rate (Table 10).

**Table 9:** Elasticities and sensitivities derived from the population projection matrices for female *Myotis nattereri*.

	Elasticity	Sensitivity
*Annual survival*		
*S*_*1*_	0.10	0.20
*S*_*2*_	0.10	0.14
*S*_*3*_	0.81	0.91
*Productivity*		
*P*_*2*_	0.00	0.03
*P*_*3*_	0.09	0.25

**Table 10:** Elasticities and sensitivities for the constituents of productivity derived from the population projection matrices for female *Myotis nattereri*.

	Vital rate	Elasticity	Sensitivity
Mean litter size in second year	*L*_*2*_	0.00	0.00
Mean litter size in third year plus	*L*_*3*_	0.09	0.09
Proportion breeding in second year	*Alpha*_*2*_	0.00	0.02
Proportion breeding in third year plus	*Alpha*_*3*_	0.09	0.13

**Fig 6:**
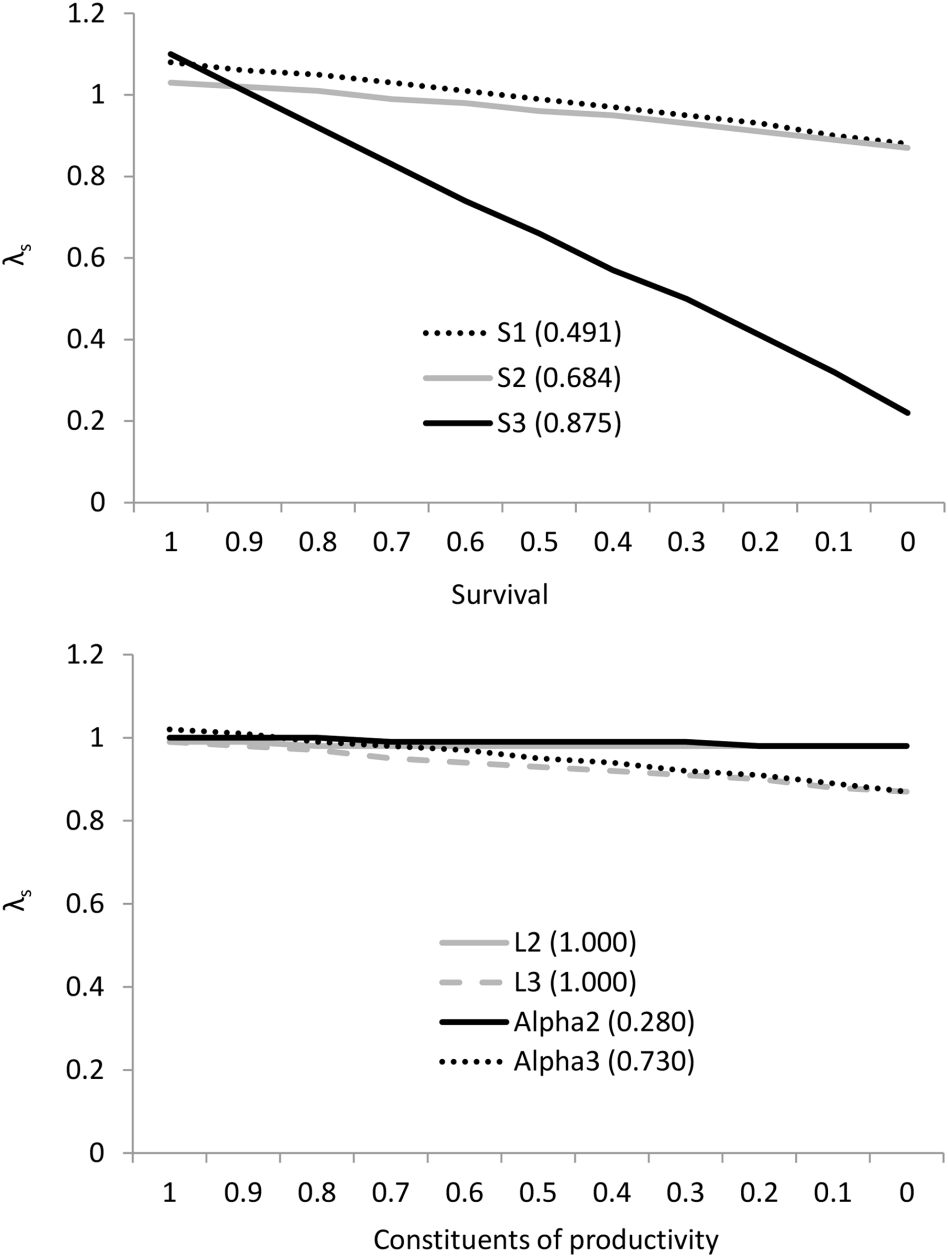
Effects of changing age-specific annual survival rates (top) and the constituents of productivity (bottom) on the population growth rate of *Myotis nattereri*. The vital rates used are shown in brackets. In the absence of perturbation, the mean stochastic growth rate λ_s_ was 0.986 i.e. a slow decline but close to stable.

To investigate the influence of perturbations in vital rates, we altered the annual survival rates (*S*_*1*_, *S*_*2*_ and *S*_*3*_), annual productivity (*P*_*2*_ and *P*_*3*_) and the constituents of productivity (*L*_*2*_, *L*_*3*_, *Alpha*_*2*_ and *Alpha*_*3*_), keeping the other rates constant to examine how changes in each of these rates would influence the population growth rate λ_s_ (Fig 6) and to calculate the threshold at which a population of 100 females is likely to become extinct (extinction probability of 1) within 500 years (Table 11).

**Table 11:** Critical threshold of population parameters for female *Myotis nattereri*, below which a population of 100 females is likely to become extinct within an arbitrary 500 years. The figures in brackets show the vital rates used in the population models.

	Vital rate	Critical values (vital rates)
*Annual survival*		
Survival in first year	*S*_*1*_	0.44 (0.491)
Survival in second year	*S*_*2*_	0.61 (0.684)
Survival in third year plus	*S*_*3*_	0.86 (0.875)
*Productivity*		
Mean litter size in second year	*L*_*2*_	negligible effect (1.000)
Mean litter size in third year	*L*_*3*_	0.92 (1.000)
Proportion breeding in second year	*Alpha*_*2*_	negligible effect (0.280)
Proportion breeding in third year plus	*Alpha*_*3*_	0.67 (0.730)

With a starting population of 100 females, with all other parameters remaining constant, annual survival would need to decline by 5% (49% to 44%) for individuals less than a year old (*S*_*1*_) to bring about population extinction (extinction probability = 1) over an arbitrary 500 year period, by 7% (68% to 61%) for individuals in their second year (*S*_*2*_), or by 2% (88% to 86%) for individuals in their third year or older (*S*_*3*_). In terms of the constituents of productivity, mean litter size of individuals breeding in their second year (*L*_*2*_) and the proportion of individuals breeding in their second year (*Alpha*_*2*_) are expected to have negligible effect on the population growth rate. Mean litter size of individuals breeding in their third year plus (*L*_*3*_) and the proportion of individuals breeding in their third year plus (*Alpha*_*3*_) would need to decline by 0.08 (1.00 to 0.92 young) and 6% (73% to 67%), respectively (Table 11).

While the number of years of simulation here is arbitrary, the results highlight that demographic monitoring should focus on obtaining robust estimates for adult survival, with a lower priority on obtaining robust estimates of first and second year survival, mean litter size of bats in their third year plus, and the proportion of individuals breeding in their third year plus.

## Discussion

Mitigating problems caused by bats in historic churches poses considerable challenges. Ensuring a sustainable future for both natural and cultural heritage will require active and creative solutions. Our data show that high intensity ultrasound, and artificial lighting if used with extreme caution, can deter bats from roosting and flying in sensitive areas of churches and limit the spread of droppings and urine that can cause nuisance and damage to the historic fabric of buildings and items of cultural significance [5].

Inside churches, *M*. *nattereri* makes use of multiple roosts, typically among exposed roof timbers, and bats move between roosts frequently. In this study, bats rarely roosted outside churches. When they did, they typically roosted alone in trees close to foraging grounds. Of nearly 2000 day roost records (*n* = 247 bats), we never recorded a bat roosting in more than one church, even when we radio-tracked bats from neighbouring churches 4 km apart. Our data on foraging suggest that individual bats and colonies occupy exclusive and non-overlapping ranges, indicating territoriality. Individual bats were faithful to core foraging areas. This spatial organisation, similar to that described in [37], and fidelity to foraging sites and to churches, means that *M*. *nattereri* may struggle to relocate quickly to new roosts if excluded from churches. Also, relocation could have a detrimental effect on foraging behaviour if bats are required to establish new foraging areas. Our modelling suggests that if excluding bats from churches resulted in reduced productivity, as for big brown bats *Eptesicus fuscus* [38], population growth may be reduced subsequently. Moreover, exclusion may result in bats becoming energetically stressed, which could affect survival, and our modelling suggests that a small reduction in adult survival could have a negative impact on population growth.

We conclude that, in the circumstances of our study, excluding *M*. *nattereri* from churches is likely to be detrimental to the welfare and Favourable Conservation Status of the species. Excluding bats from historic church buildings will, in any case, prove extremely difficult and costly, since there are often numerous access points and roosting opportunities. Identifying all access points in a church can be problematic, and bats may readily use alternative routes after major access points are blocked. Managing ‘how’ bats use churches is likely to lead to more fruitful and mutually beneficial outcomes, and may be economically preferable to attempted exclusion, given the costs of sealing churches effectively against bats.

Acoustic deterrence has considerable value as a tool for moving bats humanely from specific locations inside churches to prevent accumulations of droppings and urine below roosts, since these can damage items of cultural significance, such as historic monuments, wall paintings, and memorial brasses [5]. In our experiments the Deaton device was switched off during the day to prevent audible emissions disturbing visitors to churches. Given that bats roost mainly during the day, the effect of this deterrent may be significantly stronger if frequencies audible to humans were removed by filtering so that the devices could be switched on permanently. Our results with the CR device suggest that it should be feasible to create an effective acoustic deterrent in a form that is practical and affordable for use in churches. Further investment in, and development of, low cost ultrasonic deterrents with higher duty cycles and higher intensity will be valuable.

When the Deaton device was used at Holme Hale and Toftrees, large numbers of bats moved away from the church. These churches were notable for being comparatively small buildings occupied by large *M*. *nattereri* colonies (>130 bats), and so bats were probably not able to find suitable alternative roost locations within the churches that were sufficiently distant from the deterrent. At all other churches, most bats used an alternative roost inside the church. In the future, prior knowledge of the roosting behaviour of bats will be important to gauge the most appropriate level of acoustic deterrence to use inside churches on a case-by-case basis to avoid potential negative effects of exclusion.

We recorded only limited evidence of occupancy of bat boxes. If multiple roosts already exist in churches, and these roosts have been used historically by bats, new bat box installations are unlikely to be used preferentially. Indeed, it may take years rather than days before boxes are used to any great extent, as has been shown for *P*. *pygmaeus* in Norway [39], and so the benefits of bat boxes in short-term mitigation strategies may be limited. Encompassing major access points into a church within bespoke boxes fitted internally within churches is likely to prove more useful, as bats entering churches will enter the boxes directly. This approach will be useful in allowing bats to continue to roost within the fabric of the building while preventing access to the internal spaces, where conflict between bats and humans is typically most acute [40].

Flight activity of bats can be reduced considerably in large areas of churches by raising ambient light levels with controlled use of lighting. Use of artificial light in this way can help limit the spread of droppings and urine in churches, and so reduce cleaning burdens. Importantly, light-spill can affect the emergence behaviour of *M*. *nattereri* at roosts left in relative darkness and, if sustained, could have a negative impact on welfare by constraining foraging. When roosts are illuminated directly with artificial light, *M*. *nattereri* can become entombed. In Germany, thousands of greater mouse-eared bats *Myotis myotis* were entombed in roosts and died after lights were left on inside a church, and Bechstein’s bats *Myotis bechsteinii* roosting in mines showed a reluctance to exit roosting sites that were illuminated [Karl Kugelschafter, unpublished data], indicating that extreme aversion to light is a behavioural response shared among *Myotis* spp. Illuminating roosts and roost entrances directly, therefore, either at churches or at other roost sites, poses a serious threat to bats and so would be considered illegal in the UK without a licence. With further research into different light-types, appropriate light intensities, and effective baffles to control light spill, it may be possible to develop lighting strategies that can reduce flight activity of *M*. *nattereri* in sensitive parts of churches without adversely effecting roosting and emergence behaviour.

*Pipistrellus* spp. may be less deterred by lights [14,41]. In this study, passes by common pipistrelles *P*. *pipistrellus* and soprano pipistrelles *P*. *pygmaeus* reduced substantially in illuminated parts of churches when lights were switched on, but neither species was deterred completely. At one church that we surveyed but did not include in the experimental trials, we observed hundreds of *P*. *pygmaeus* flying inside the building when internal security lights were left on at night, suggesting that this species can habituate to lights. Future research should focus on examining the responses of other species to both acoustic and light deterrence, as responses are likely to be species-specific, given inter-specific differences in audition and light tolerance.

Bats show high fidelity to nursery roosts [21,42,43]. We found that *M*. *nattereri* moves between roosts more frequently in spring, and moves from roosts more readily in response to deterrence, than when juvenile bats are present. It may be preferable, therefore, to deter bats from roosts in the spring when they are more transient, so long as alternative roosts are known to be available. This could help to reduce the level of deterrence required to move bats and minimise disturbance caused at roosts, and in particular to naïve juvenile bats that may have limited knowledge of alternative roosts.

## Conclusions

Many bat colonies in churches are of considerable conservation value, and their protection, together with the provision of access to churches as places of worship, and the protection of irreplaceable cultural artefacts, must all be valued highly. We predict that excluding *M*. *nattereri* from churches is likely to have a negative impact on welfare and Favourable Conservation Status, at least in situations where few suitable alternative roosts exist. Even so, with judicious use of deterrents, problems caused by bats in churches can be mitigated. Deterrents can be used to move roosting sites within churches and limit the spread of droppings and urine so that problems to congregations and to artefacts of historic and cultural significance can be greatly reduced. While we provide proof of concept that these deterrents can be effective, none was designed specifically for use in churches, and so further development of these tools will be necessary to make them more practical and to ensure that they are as safe as possible for bats. Deterrence has the potential to cause serious harm to bats if used inappropriately, and so the use of deterrents will need to be strictly regulated to protect Favourable Conservation Status. A ‘carrot and stick’ approach, creating roosting spaces that limit access to church interiors, in combination with acoustic deterrents that keep bats away from areas where problems are most severe, has great potential for mitigating damage caused by bats in this classic case of human-wildlife conflict.

## Supporting Information

S1 Appendix Description of bespoke roost spaces created at St. Andrew’s church, Holme Hale, Norfolk. (DOCX)

S2 Appendix Additional explanation of population modelling procedures. (DOCX)

S1 Table Description of the habitat types used in compositional analysis to determine the habitat preferences of radio-tracked *Myotis nattereri*. (DOCX)

S2 Table Results from multistate models to examine if the roosting behaviour of radio-tagged Myotis nattereri was affected significantly by short-term applications of acoustic deterrence (Deaton device) at roosts inside churches. Model 1: comparing two states, original roost vs other roost, with response being a roost change. Model 2: comparing two states, inside church vs outside church, with response being a roost change.
(DOCX)
