# Correction: Mitigating the Impact of Bats in Historic Churches: The Response of Natterer's Bats *Myotis nattereri* to Artificial Roosts and Deterrence

**DOI:** 10.1371/journal.pone.0152531

**Published:** 2016-03-24

**Authors:** Matt R. K. Zeale, Emily Bennitt, Stuart E. Newson, Charlotte Packman, William J. Browne, Stephen Harris, Gareth Jones, Emma Stone

One affiliation for the second author is not indicated. Emily Bennitt is also affiliated with: Okavango Research Institute, University of Botswana, Private Bag 285, Maun, Botswana. The affiliations should appear as shown here:

Matt R. K. Zeale^1^, Emily Bennitt^1,2^, Stuart E. Newson^3^, Charlotte Packman^1^, William J. Browne^4^, Stephen Harris^1^, Gareth Jones^1^*, Emma Stone^1^

**1** School of Biological Sciences, Life Sciences Building, University of Bristol, 24 Tyndall Avenue, Bristol, BS8 1TQ, United Kingdom, **2** Okavango Research Institute, University of Botswana, Private Bag 285, Maun, Botswana, **3** British Trust for Ornithology, The Nunnery, Thetford, Norfolk, IP24 2PU, United Kingdom, **4** Graduate School of Education, and Centre for Multilevel Modelling, University of Bristol, 2 Priory Road, Bristol, BS8 1TX, United Kingdom
