# Supplementary material for: Mitigating the Impact of Bats in Historic Churches: The Response of Natterer’s Bats Myotis nattereri to Artificial Roosts and Deterrence
Source: PLoS One. 2016 Jan 15;11(1):e0146782. doi: 10.1371/journal.pone.0146782 (PMC4714818; doi:10.1371/journal.pone.0146782)

**S1 Appendix: Description of bespoke roost spaces created at St. Andrew’s church, Holme Hale, Norfolk.**

Bespoke roost spaces were created by installing hardwood boards inside the church to ‘box in’ existing roosts in mortise joints used by *Myotis nattereri*. Two separate roost spaces were created, each encompassing a major entry point used by bats accessing the interior of the church. The entry points remained unaltered i.e. bats were able to enter the church as normal, but on entry bats were contained within a large roost void approximately five metres in length that was sealed off from the rest of the internal space of the church where previously the deposition of droppings and urine caused significant problems. Each roost space was sufficiently capacious to allow bats to fly within and to accommodate the entire breeding colony of over 130 bats.


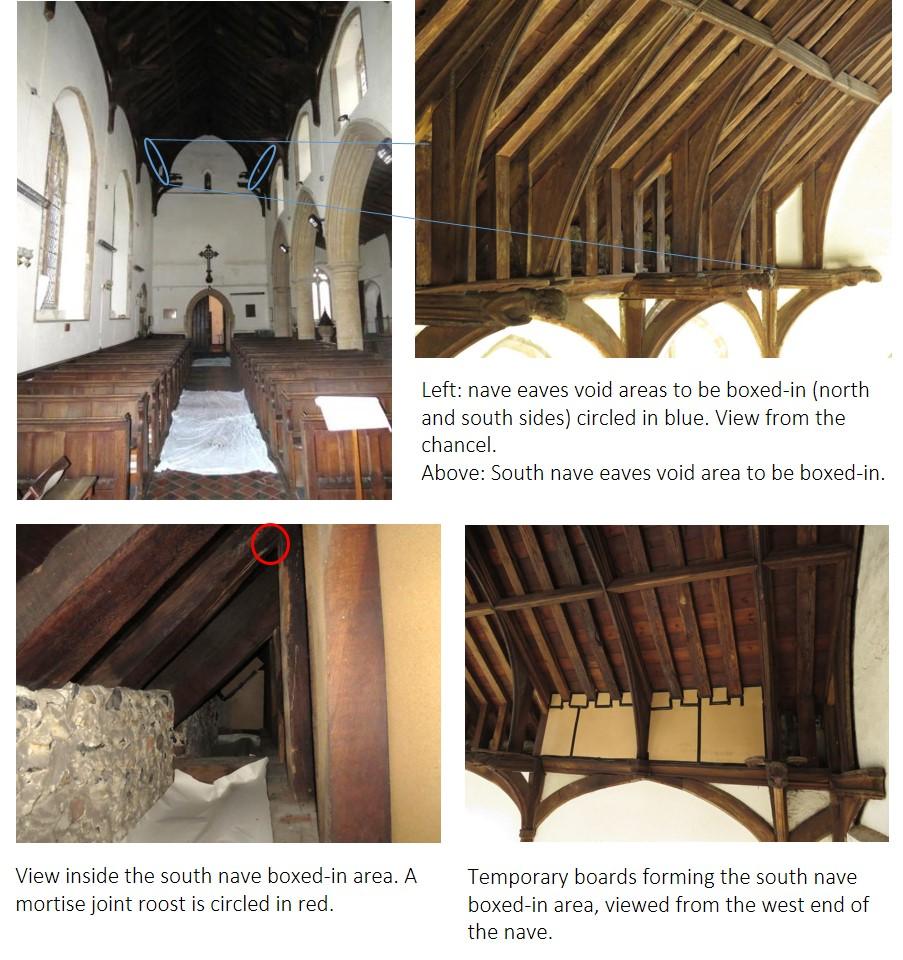

Supplement: S1 Appendix — (DOCX) [file pone.0146782.s001.docx]
