# Supplementary material for: Mitigating the Impact of Bats in Historic Churches: The Response of Natterer’s Bats Myotis nattereri to Artificial Roosts and Deterrence
Source: PLoS One. 2016 Jan 15;11(1):e0146782. doi: 10.1371/journal.pone.0146782 (PMC4714818; doi:10.1371/journal.pone.0146782)
Supplement: S2 Appendix — (DOCX) [file pone.0146782.s002.docx]

**S2 Appendix: Additional explanation of population modelling procedures.**

For our analyses, population size *n* was defined as the number of females in a population and was the sum of the numbers in each age class

*n = n_1_ + n_2_ + n_3_ …. n_i_* (1)

where n*_i_* is the number of females in age class *i*. A population with three age classes can be described by a set of difference equations, where *P_i_* are age-specific productivities i.e. the number of female young produced at each age class *i* and *Si* are age-specific survival probabilities of age class *i*, such that

*n*_1_(*t*+1) = *P*_2_*n*_2_(*t*) + *P*_3_*n*_3_(*t*)

*n*_2_(*t*+1) = *S*_1_*n*_1_(*t*) (2)

*n*_3_(*t*+1) = *S*_2_*n*_2_(*t*) + *S*_3_*n*_3_(*t*)

So a generic age structured bat population can be represented as a life-cycle graph thus

The difference equations describing the bat population can be written more simply in matrix form

**n**(*t*+1) = **An**(*t*), (3)

where **n** is an age distribution vector

**n**(*t*) =$\left[\begin{matrix} \text{ }n_{1}\text{(t) } \\ \text{ n}_{2}\text{(t)} \\ \text{ n}_{3}\text{(t)} \end{matrix}\right]$, (4)

and **A** is a population projection matrix as described by [1,2]. The elements of the matrix can be obtained from the difference equations or from the life-cycle graph, so that

= **$\left[\begin{matrix} P_{1} & P_{2} & P_{3} \\ S_{1} & 0 & 0 \\ 0 & S_{2} & S_{3} \end{matrix}\right]$** (5)

This is known as an extended Leslie matrix [1,2]; it assumes the vital rates in the matrix are equal after age class two.

By repeatedly multiplying the matrix **A** by the vector **n**, a series of vectors are obtained that stabilise out after a few generations, so they differ from each other by a scalar factor. This factor λ, is the dominant eigenvector of the population projection matrix **A**, and is equal to the population growth rate, so that

**n**(*t*+1) = λ**n**(*t*), (6)

where

λ = *e^r^* , (7)

*r* being the per capita rate or intrinsic rate of population growth. Successively multiplying **n** by **A** also results in the vector **w** that is proportional to the stable age structure of the population.

**w** =$\left[\begin{matrix} \text{ }w_{1}\text{ } \\ \text{ }w_{2} \\ \text{ }w_{3} \end{matrix}\right]$, (8)

The stable age structure **w** and λ can be used to determine the population size **n** at any time *t*, thus

**n**(*t*) = λ*^t^***w**. (9)

Substituting this into (3) gives

λ^t+1^**w** = λ*^t^***Aw**. (10)

A scalar λ and vector **w** that satisfy this equation are the dominant eigenvalue and right eigenvector of the matrix **A**, respectively. These must satisfy

(**A** - λ**I**) **w** = 0, (11)

where **I** is the identity matrix. **w** has a nonzero solution only if the determinant of the matrix.

**A** - λ**I** equals zero, hence the value of λ and **w** can be solved using the characteristic equation

det (**A** - λ**I**) = 0, (12)

The characteristic equation can be solved using polynomial expansion, which for bats here with a matrix of three by three, produces seven eigenvalues and their associated eigenvectors, where λ is the dominant eigenvalue. This also produces a solution for the left eigenvector **v** of the matrix **A** which is known as the reproductive stage vector and represents the contribution of each age class to the population, so that

**v**^*^**A** = λ**v**^*^, (13)

where **v**^*^ is the transpose of **v**. The left and right eigenvectors can be scaled so that their scalar product equals one, so for the eigenvalue λ

〈**w,v**〉 = **v**^*^**w** = 1, (14)

where 〈**w,v**〉 is the scalar product of **w** and **v**. This allows calculation of the effect that a small additive change in any of the vital rates of the population projection matrix **A** would have on λ, so that

$s_{ij}=\frac{\partial\lambda}{\partial\text{a}_{\text{ij}}}=v_{i}w_{j},$ (15)

where s_ij_ is the sensitivity of λ to changes in the matrix element a*_ij_*. The sensitivity of λ to changes in other parameters can then be calculated for any other vital rate (*x*).

$\frac{\partial\lambda}{\partial\text{ x}}=\sum_{i,j} \frac{\partial\lambda}{\partial\text{a}_{\text{ij}}}\frac{\partial\text{a}_{\text{ij}}}{\partial\text{x}}.$ (16)

Often more important in conservation and management is to determine the proportional effect that a small change in each vital rate would have on λ. This can be examined using elasticity analysis, where

$e_{ij}=\frac{a_{ij}}{\lambda}\frac{\partial\lambda}{\partial\text{a}_{\text{ij}}}.$ (17)

so the elasticity to other parameters can be calculated by

$e(x)=\frac{x}{\lambda}\frac{\partial\lambda}{\partial x}$ (18)

$\text{ }=\frac{x}{\lambda}\sum_{i,j} \frac{\partial\lambda}{\partial a_{ij}}\frac{\partial a_{ij}}{\partial x}.$ (19)

**Estimating vital rates for Natterer’s bat**

Productivity is assumed to be 1 for *M. nattereri*. Twins have been recorded but the level of twinning in *M. nattereri* in the British Isles is believed to be negligible [3]. We assume an equal sex ratio of females at birth, and that female bats can start breeding at one year of age, such that

$P_{i}=L_{i}*alpha_{i}$ (20)

where *P_i_* is the productivity of age class *i* (where *i* = {1, 2}), *L_i_* is the mean litter size and *alpha_i_* is the proportion of age class *i* breeding*.* The productivity of bats in their first year (i.e. infants) will be 0. In relation to the proportion of individuals breeding in any given year, our data recorded from *M. nattereri* maternity roosts during this study show that 170 of 233 adult females (73%) and 5 of 18 first year females (28%) showed signs of pregnancy, lactation or post-lactation. We used these as an estimate of the likely number of individuals of each age-class breeding, with the caveat that these figures may underestimate the true proportion of each age-class breeding because it is difficult on occasions to confirm in the hand whether a bat has bred. Productivity was expressed as

$\begin{matrix} P_{1}=0 \\ \\ P_{2}=L_{2}*alpha_{2} \\ \\ P_{3}=L_{3}*alpha_{3} \end{matrix}$ (21)

We use estimates of first year, second year and adult survival in [4]. An estimate of female adult survival for Natterer's bat produced in [5] is comparable with that recorded in [4].

**References**

1. Leslie PH (1945) On the use of matrices in certain population mathematics. Biometrika 33: 183-212. doi: 10.2307/2332297

2. Leslie PH (1948) Some further notes on the use of matrices in population mathematics. Biometrika 35: 213-245. doi: 10.1093/biomet/35.3-4.213

3. Smith PG, Rivers NM. Natterer’s bat *Myotis nattereri*. In: Harris S, Yalden DW, editors. Mammals of the British Isles: Handbook. 4th edition. Southampton: The Mammal Society; 2008. pp. 323-328.

4. Mortimer G. Foraging, roosting and survival of Natterer’s bats *Myotis nattereri* in a commercial coniferous plantation. Ph.D. Thesis, University of St. Andrews. 2006.

5. Rivers NM, Butlin RK, Altringham JD (2006) Autumn swarming behaviour of Natterer’s bats in the UK: population size, catchment area and dispersal. Biol Conserv 127: 215-226. doi: doi:10.1016/j.biocon.2005.08.010
