# Supplementary material for: Mitigating the Impact of Bats in Historic Churches: The Response of Natterer’s Bats Myotis nattereri to Artificial Roosts and Deterrence
Source: PLoS One. 2016 Jan 15;11(1):e0146782. doi: 10.1371/journal.pone.0146782 (PMC4714818; doi:10.1371/journal.pone.0146782)
Supplement: S1 Table — (DOCX) [file pone.0146782.s003.docx]

**S1 Table: Description of the habitat types used in compositional analysis to determine the habitat preferences of radio-tracked *Myotis nattereri*.**

| Habitat | Description |
| --- | --- |
| Arable | Ploughed land, cropland and recently reseeded grassland. Includes arable land and grassland in rotation, horticultural land and nurseries, and recently planted and established orchards. |
| Grassland | Any grassland not included under riparian. Includes improved, semi-improved and unimproved types, enclosed meadows and pastures, and amenity grasslands. |
| Riparian | Open water and marginal vegetation around any water body, including rivers, streams, brooks, lakes, ponds (including operational ponds), reservoirs, aquaculture, estuary and coastal waters, riparian woodland, wet heathland, tall vegetation along water courses, swamp vegetation around pools and all types of fen and mire. |
| Built-up | Roads, houses and residential land, built-up areas, including areas of commercial retail, industry, high density residential (>40% cover), agricultural buildings, transport areas, restored or active landfill sites, and active or inactive quarries. |
| Woodland | Any woodland not included under riparian. Includes broadleaved, conifer and mixed types, ancient and young stands, forestry scrub, and encompassing all management types including plantation, restoration, coppice, minimum intervention, etc. |
