# Supplementary material for: Mitigating the Impact of Bats in Historic Churches: The Response of Natterer’s Bats Myotis nattereri to Artificial Roosts and Deterrence
Source: PLoS One. 2016 Jan 15;11(1):e0146782. doi: 10.1371/journal.pone.0146782 (PMC4714818; doi:10.1371/journal.pone.0146782)
Supplement: S2 Table — Model 1: comparing two states, original roost vs other roost, with response being a roost change. Model 2: comparing two states, inside church vs outside church, with response being a roost change. (DOCX) [file pone.0146782.s004.docx]

**S2 Table: Results from multistate models to examine if the roosting behaviour of radio-tagged *Myotis nattereri* was affected significantly by short-term applications of acoustic deterrence (Deaton device) at roosts inside churches.** Model 1: comparing two states, original roost vs other roost, with response being a roost change. Model 2: comparing two states, inside church vs outside church, with response being a roost change.

|  |  |  |  |  |
| --- | --- | --- | --- | --- |
| Move to  (fixed part) | Period | Single probs (SE) | Diff prob move to original (SE) | Diff prob both states (SE) |
|  |  |  |  |  |
|  |  |  |  |  |
| *Model 1* |  |  |  |  |
|  |  |  |  |  |
| Original roost | Control | -1.867 (0.157) | -0.047 (0.305) | -0.047 (0.305) |
| Original roost | Deterrent |  | -4.020 (0.657) | -4.020 (0.657) |
| Original roost | Post-deterrent |  | -1.491 (0.382) | -1.491 (0.382) |
|  |  |  |  |  |
| Other roost | Control | -1.153 (0.130) | -1.153 (0.130) | -2.730 (0.297) |
| Other roost | Deterrent |  |  | 3.246 (0.363) |
| Other roost | Post-deterrent |  |  | 0.858 (0.614) |
|  |  |  |  |  |
| *P value* |  |  | <0.0001 | <0.0001 |
|  |  |  |  |  |
| *Model 2* |  |  |  |  |
|  |  |  |  |  |
| Inside church | Control | -0.936 (0.187) | -0.000 (0.343) | 0.000 (0.343) |
| Inside church | Deterrent |  | -1.668 (0.486) | -1.668 (0.486) |
| Inside church | Post-deterrent |  | -0.901 (0.475) | -0.901 (0.475) |
|  |  |  |  |  |
| Outside church | Control | -2.657 (0.173) | -2.657 (0.175) | -2.970 (0.324) |
| Outside church | Deterrent |  |  | 0.854 (0.392) |
| Outside church | Post-deterrent |  |  | -1.064 (0.784) |
|  |  |  |  |  |
| *P value* |  |  | 0.0028 | 0.0077 |
|  |  |  |  |  |
